# Fingolimod Limits Acute Aβ Neurotoxicity and Promotes Synaptic Versus Extrasynaptic NMDA Receptor Functionality in Hippocampal Neurons

**DOI:** 10.1038/srep41734

**Published:** 2017-01-30

**Authors:** Pooja Joshi, Martina Gabrielli, Luisa Ponzoni, Silvia Pelucchi, Matteo Stravalaci, Marten Beeg, Sonia Mazzitelli, Daniela Braida, Mariaelvina Sala, Enrica Boda, Annalisa Buffo, Marco Gobbi, Fabrizio Gardoni, Michela Matteoli, Elena Marcello, Claudia Verderio

**Affiliations:** 1 IRCCS Humanitas, via Manzoni 56, 20089 Rozzano, Italy; 2 CNR Institute of Neuroscience, via Vanvitelli 32, 20129 Milano, Italy; 3 Department of Medical Biotechnology and Translational Medicine, Università degli Studi di Milano, via Vanvitelli 32, 20129 Milano, Italy; 4 Fondazione Umberto Veronesi, Milano, Italy; 5 Department of Pharmacological and Biomolecular Sciences, University of Milan, via Balzaretti 9, 20133 Milan, Italy; 6 Department of Molecular Biochemistry and Pharmacology IRCSS – Istituto di Ricerche Farmacologiche Mario Negri, via La Masa 19, 20156 Milan, Italy; 7 Department of Neuroscience Rita Levi-Montalcini, University of Turin, 10126, Turin, Italy

## Abstract

Fingolimod, also known as FTY720, is an analogue of the sphingolipid sphingosine, which has been proved to be neuroprotective in rodent models of Alzheimer’s disease (AD). Several cellular and molecular targets underlying the neuroprotective effects of FTY720 have been recently identified. However, whether the drug directly protects neurons from toxicity of amyloid-beta (Aβ) still remains poorly defined. Using a combination of biochemical assays, live imaging and electrophysiology we demonstrate that FTY720 induces a rapid increase in GLUN2A-containing neuroprotective NMDARs on the surface of dendritic spines in cultured hippocampal neurons. In addition, the drug mobilizes extrasynaptic GLUN2B-containing NMDARs, which are coupled to cell death, to the synapses. Altered ratio of synaptic/extrasynaptic NMDARs decreases calcium responsiveness of neurons to neurotoxic soluble Aβ 1–42 and renders neurons resistant to early alteration of calcium homeostasis. The fast defensive response of FTY720 occurs through a Sphingosine-1-phosphate receptor (S1P-R) -dependent mechanism, as it is lost in the presence of S1P-R1 and S1P-R3 antagonists. We propose that rapid synaptic relocation of NMDARs might have direct impact on amelioration of cognitive performance in transgenic APPswe/PS1dE9 AD mice upon sub-chronic treatment with FTY720.

Aggregates of amyloid beta (Aβ) in the brain parenchyma and deposits of hyperphosphorylated tau in neurons are hallmarks of Alzheimer’s disease(AD), the most common neurodegenerative disorder characterized by synaptic dysfunction, neuronal loss and cognitive impairment. Consolidated evidence indicates that soluble Aβ forms and tau species rather than insoluble aggregates are responsible for neuronal damage and cognitive decline^1^,^2^,^3^,^4^,^5^. While molecular mechanisms mediating neurotoxicity of soluble tau forms largely remain to be elucidated, glutamate ionotropic NMDA receptors (NMDARs) have emerged as specific targets of soluble Aβ 1–42 (s-Aβ) oligomers. NMDARs are among surface molecules which mediate s-Aβ interaction with neurons and evidence has been recently provided for a causal role of s-Aβ binding to or near NMDARs and neuronal damage^1^,^6^,^7^,^8^.

NMDARs are distinguished by localization and subunit composition in two functional distinct pools, which differentially regulate neuronal activity and survival^9^. Synaptic GLUN2A-containing NMDARs are neuroprotective and involved in plasticity phenomena. Extrasynaptic GLUN2B-containing NMDARs are coupled to cell death^10^,^11^ and implicated in neurodegenerative disorders^12^,^13^. S-Aβ has been extensively demonstrated to preferentially activate extrasynaptic GLUN2B-containing receptors^14^,^15^,^16^, although it elicits inward currents in both GLUN2A- and GLUN2B-containing NMDARs, when heterologously expressed in *Xenopus laevis* oocytes^7^.

Fingolimod, also known as FTY720, is an oral immunosuppressant, successfully used to treat multiple sclerosis^17^,^18^. It is an analogue of the sphingolipid sphingosine, and upon phosphorylation by sphingosine kinase 2 acts as sphingosine-1-phosphate (S1P) agonist on all S1P receptors (S1P-Rs), except S1P-R2^19^. The therapeutic action of FTY720 in multiple sclerosis is mainly mediated by S1P-R1, a receptor which become internalized upon binding of FTY720-P. Decrease in functional S1P-R1s prevents egress of autoagressive T cells from lymph nodes and autoimmune brain reaction^20^,^21^,^22^. Besides this peripheral action, FTY720 readily penetrates the CNS of rodents^23^,^24^ and humans^25^ and exerts protective effects on brain cells, including oligodendrocytes^26^, astrocytes^27^, microglia^28^,^29^ and neuron^30^,^31^,^32^. How the drug acts on neurons is not yet well understood, although neurons express S1P-Rs^23^,^31^,^33^,^34^,^35^ and thus may be a direct cellular target of FTY720.

Thanks to its broad positive action on brain cells, FTY720 is emerging as promising neuroprotective agent in a wide range of CNS diseases. It exerts therapeutic benefit in preclinical models of stroke^36^,^37^,^38^ trauma^39^, Rett Syndrome^40^, epilepsy^41^ and also AD^31^,^42^,^43^,^44^. In rodents models of AD, i.e. rats or mice injected with Aβ, FTY720 ameliorates memory impairment^43^,^44^,^45^, while *in vitro* it protects neurons from s-Aβ toxicity^31^,^32^. Multiple mechanisms have been implicated in the protective action of FTY720 in AD, including inhibition of Aβ production from neurons^42^, modulation of microglia activation and cytokine release^30^, regulation of the ceramide/S1P balance^46^ and up regulation of neuronal brain-derived neurotrophic factor (BDNF)^31^,^40^,^43^ a key modulator of memory formation^47^. Intriguingly, upregulation of the growth factor may be independent of S1P-R activation, resulting from nuclear action of FTY720, which inhibits histone deacetylases and exerts epigenetic control on genes associated to learning and memory^24^, similarly to S1P.

In this study we suggest a new mechanism underlying direct protective action of FTY720 on neurons. We propose that the drug acutely protects neurons from s-Aβ toxicity by enhancing the functionality of synaptic versus extrasynaptic NMDARs through a S1PR-dependent mechanism. By this pathway the drug may contribute to amelioration of cognitive impairment in transgenic APPswe/PS1dE9 AD mice upon subchronic administration.

## Results

### Subchronic treatment with FTY720 improves memory performance and reduces s-Aβ concentration in APPswe/PS1dE9 transgenic mice

Previous evidence indicates that FTY720 ameliorates impairment in spatial memory and associative learning in rat or mice injected with Aβ^43^,^44^. To explore the therapeutic potential of FTY720 in a transgenic AD mouse model, FTY720 (1 mg/Kg) or plain water was administered by oral gavage to 12 months-old APPswe/PS1dE9 and their littermates for 6 weeks. APPswe/PS1dE9 mice develop first Aβ plaques at 4 months of age and clear cognitive defects at 12 months^48^. These mice do not exhibit neuronal loss, but display clinically relevant AD-like symptoms such as gliosis and microgliosis and correlation of the s-Aβ levels with behavioural deficits^49^. Pre-drug and post-drug behavioural analysis was carried out to test learning ability and different forms of memory performance, i.e., reference, episodic and innate memory, using the passive avoidance^50^ the novel object recognition^51^ and the nest building tasks (Zhe *et al*., 2013), respectively (Supplementary Fig. 1A–E). As expected, 12 months-old male APPswe/PS1dE9 mice showed basally (one week before treatment) (Supplementary Fig. 1B) severe deficits in reference memory in the passive avoidance task, in terms of reduced latency to re-enter in the dark compartment, where mice receive a mild foot shock (Supplementary Fig. 1B). After 5 weeks treatment with plain water their performance was further worsened. Conversely, their cognitive performance was significantly preserved upon treatment with FTY720 (Supplementary Fig. 1B, left). The novel object recognition memory task showed similar findings, revealing a reduced discrimination index during basal recordings and a further worsening in vehicle-treated APPswe/PS1dE9 mice. An improved recognition index was observed in FTY720-treated double transgenic mice (Supplementary Fig. 1C). Female APPswe/PS1dE9 mice were partially protected from memory impairment (Supplementary Fig. 1B). They were never impaired in the passive avoidance task, but became impaired in the novel object recognition task after 5 weeks of treatment with plain water, while maintained performance similar to wild type littermates upon FTY720 administration (Supplementary Fig. 1C). Measurements of basal locomotor activity excluded motor interference in the cognitive tasks. Indeed male mice, which were impaired in both cognitive tasks, showed normal motor activity while female mice were slightly hyperactive (Supplementary Fig. 1D). Consistent with a therapeutic action of FTY720, in the nest building task, there was an improvement of nest building score in APPswe/PS1dE9 mice compared to corresponding vehicle-treated group (Supplementary Fig. 1E). In this task, no major difference in the behavior of male and female APPSwe/PS1 mice was observed.

We next examined the impact of subchronic FTY720 treatment on brain inflammation and Aβ content, the two clinically relevant AD-like symptoms of APPswe/PS1dE9 mice. A trend to decrease in the astrogliosis marker GFAP was observed by western blot analysis in APPswe/PS1dE9 mice administered with FTY720, while no changes in the microglial marker IBA-1 were detected in AD mice upon FTY720 treatment (not shown). Conversely, dot blot analysis of proteins extracted from the cortex of APPSwe/PS1 mice with anti-Aβ 6E10 antibody showed a significant decrease in Aβ 1–42 levels in FTY720-treated mice (Supplementary Fig. 1F). In addition, Aβ1–42 Elisa revealed a significant reduction in s-Aβ isolated in detergent-free buffer from the hippocampus of APPSwe/PS1 mice that received FTY720 as compared to mice treated with plain water (Supplementary Fig. 1G). The decrease was stronger in female than male transgenic mice (not shown).

### FTY720 protects cultured neurons from acute s-Aβ toxicity

The decrease in Aβ content may account for amelioration of memory performance in the transgenic APPSwe/PS1 mouse. However, FTY720 may also render neurons less vulnerable to neurotoxic s-Aβ. We deeply investigated this hypothesis *in vitro* by exploring how FTY720 impacts the viability of hippocampal neurons exposed to toxic s-Aβ.

**Figure 1:**
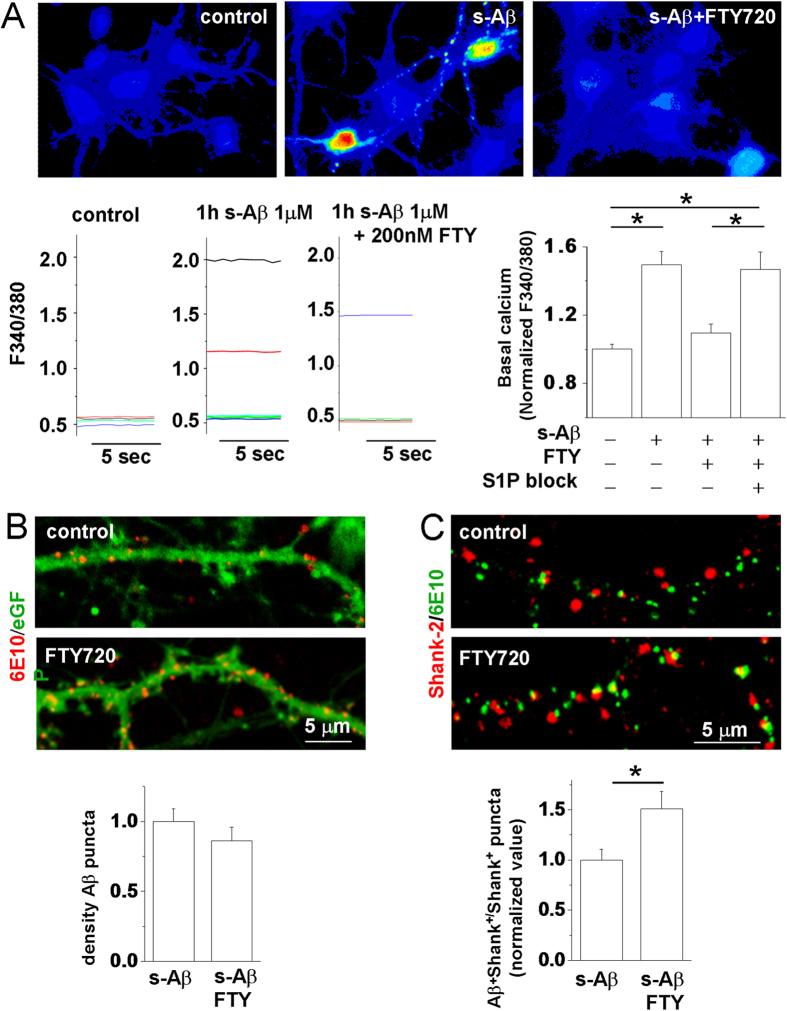
FTY720 protects neurons from s-Aβ-induced calcium dysregulation by altering the binding of s-Aβ to neurons. Basal [Ca^2+^]_i_ was measured in 9DIV neurons, loaded with the ratiometric calcium dye Fura-2 and expressed as F340/380 fluorescence. (**A**) Representative pseudocolor images of basal [Ca^2+^]_i_ in control neurons and in neurons exposed to s-Aβ alone or in combination with FTY720 for 1 h. Representative traces and quantification of basal [Ca^2+^]_i_ are shown below. Values are normalized to control (the Kruskal–Wallis ANOVA, P = 0.002; Dunn’s test for comparison among groups, P = 0.05; N = 4). (**B**) Representative confocal images of 14DIV eGFP+ mouse neurons exposed to s-Aβ alone or in combination with FTY720, fixed and stained with the anti-Aβ antibody 6E10. Quantification of Aβ binding to neurons is shown below. The histogram shows density of Aβ puncta bound to neurons, (number of Aβ/eGFP colocalizing puncta normalized over eGFP area) (Mann-Whitney Rank Sum Test, P = 0.153; N = 3). (**C**) Representative confocal images of mouse neurons treated as in (**B**) and probed for 6E10 and the postsynaptic density marker shank-2. The panel below shows the fraction of Shank-2 puncta with bound Aβ (Mann-Whitney Rank Sum Test, P = 0.043; N = 3).

Our previous evidence indicates that cultured hippocampal neurons display abnormally high levels of cytosolic calcium as early as 1 h after exposure to s-Aβ 1–42 and eventually undergo cell death (within 24 h)^32^. We took advantage of this early sign of neurodegeneration to investigate the possibility of a precocious protective effect of FTY720. We recorded cytosolic calcium from 9 DIV neurons, loaded with the calcium dye Fura-2, 1 h after exposure to 1 μM s-Aβ in the presence or in the absence of 200 nM FTY720. We found that FTY720 significantly protected neurons from early alterations of calcium homeostasis induced by s-Aβ (Fig. 1A), indicating a fast-acting protective action. Neuroprotection was completely lost in the presence of the S1P-R1 and S1P-R3 selective antagonists W146 (10 μM)^52^ and CAY1044 (10 μM)^53^,^54^ (Fig. 1A), the S1P-Rs mainly expressed in hippocampal neurons^34^,^55^.

### FTY720 enhances binding of s-Aβ to synaptic sites

We then asked whether rapid neuroprotection by FTY720 may result from alteration in the binding of s-Aβ to neurons. 14–16 DIV eGFP positive neurons, established from a transgenic mouse line ubiquitously expressing eGFP under actin promoter, were incubated with s-Aβ (1 μM) alone or in combination with 200 nM FTY720 for 1 h. Neurons were then extensively washed to remove unbound Aβ, fixed and stained with the anti-Aβ antibody 6E10. We did not observe significant changes of s-Aβ binding to eGFP positive neurites in FTY720-treated neurons (Fig. 1B). However, double staining of neurons for 6E10 and the postsynaptic protein Shank-2 showed a significant increase in the fraction of postsynaptic sites with bound Aβ under drug treatment (Fig. 1C). Similar results were obtained in rat neurons (fraction of Aβ + Shank-2 puncta -normalized values-: control = 1.009 ± 0.06, FTY720 = 1.304 ± 0.07; N = 3, Mann-Whitney Rank Sum Test, P < 0.001; Data not shown). No changes in the density of Shank-2 positive puncta were observed upon 1 h exposure to s-Aβ (number of Shank-2 + puncta/ μm: control = 0.81 ± 0.0350, s-Aβ = 0.83 ± 0.0279; number of dendrites analysed = 20; Data not shown).

### FTY720 does not bind to Aβ species

Membrane lipids, including gangliosides and sphingolipids, are known to interact with both insoluble^32^,^56^ and soluble Aβ forms^57^ and to influence Aβ neurotoxicity. Thus, we explored whether FTY720, a sphingolipid analog, may interact with Aβ species and enhance their binding to synaptic neuronal receptors.

**Figure 2:**
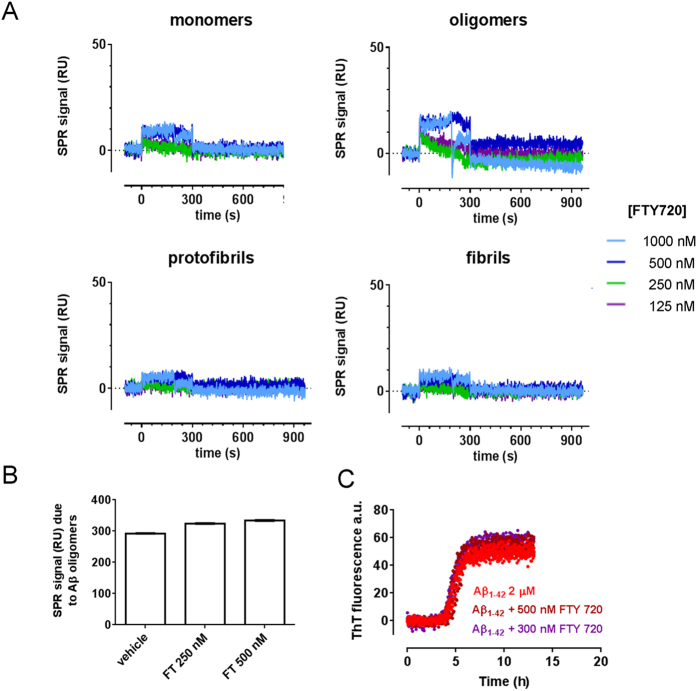
FTY720 does not interact with Aβ species and does not interfere with formation of Aβ oligomers or fibrillogenesis. (**A**) Synthetic Aβ1–42 (100 μM) was incubated in quiescent conditions for different time points in order to prepare the different Aβ species. We used freshly prepared solutions to have Aβ42 monomers only; 5 h and 24 h incubation at 25 °C for maximal oligomers and protofibrils enrichment, respectively; fibrils were prepared by 24 h incubation at 37 °C at pH 2.0. These species were immobilized on the sensor chip coated whereas FTY720 (concentrations indicated) was flowed from time = 0 to time = 300 sec. (**B**) Synthetic Aβ1–42 (100 μM) was incubated at 25 °C in the absence or presence of different concentrations of FTY720. After 5 h samples were diluted 100 fold in PBST and injected over immobilized 4G8 for 2 min, followed by 10 min of dissociation. The bars shows the SPR binding signal measured at the end of the dissociation period, which is indicative of the amount of captured Aβ oligomers. (**C**) Synthetic Aβ1–42 (2 μM) was incubated with ThT (10 μM) with or without FTY720, at two different dilutions. ThT fluorescence was continuously monitored for 13 h.

Direct interaction of FTY720 with Aβ forms was assessed by surface plasmon resonance (SPR)^58^. Briefly, Aβ monomers, oligomers, protofibrils and fibrils were immobilized on the sensor chip, and different concentrations of FTY720 (ranging from 125 to 1000 nM) were flowed over them. The resulting sensorgrams showed no relevant binding of FTY720 to Aβ species (Fig. 2A). We next investigated whether FTY720 might interfere with the formation of s-Aβ oligomeric species. In these experiments s-Aβ oligomers were recognized by their pseudo-irreversible binding to the anti-Aβ antibody 4G8 immobilized on the sensor chip^59^. Figure 2B shows that the presence of FTY720 (250–500 nM) did not affect the formation of Aβ oligomers from monomers. Finally, we monitored possible alteration in the kinetics of Aβ fibril formation using the thioflavin-T(ThT) assay. Aβ 1–42 (2 μM) was incubated in the presence of 300–500 nM FTY720 and ThT at 28 °C. Time course analysis showed that in our conditions Aβ fibrillogenesis had a lag phase of 2 h, followed by very rapid growth, reaching a plateau after 5 h (Fig. 2C), and it was not affected by FTY720 (Fig. 2C).

Collectively these data rule out direct interaction between FTY720 and Aβ species, thus excluding that changes in Aβ conformation/aggregation may account for alteration in Aβ binding to synaptic sites.

### FTY720 decreases functionality of extrasynaptic NMDARs while promotes activation of NMDARs at the synapse

**Figure 3:**
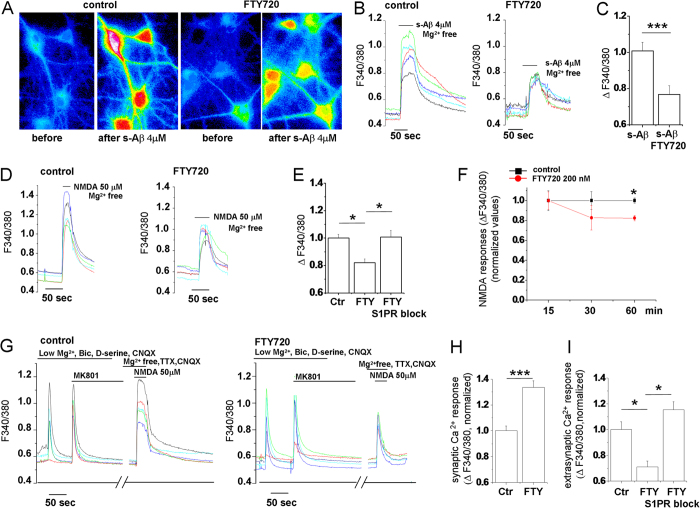
FTY720 increases the ratio between synaptic and extrasynaptic calcium-permeable NMDA receptors. (**A**) Representative pseudocolor images of Fura-2 loaded neurons (9DIV) maintained in control conditions or pre-treated with FTY720(200 nM, 1 h) before stimulation with s-Aβ (4 μM) in Mg^2+^ -free medium and at peak [Ca^2+^]_I_ responses. (**B**) Representative temporal plots of [Ca^2+^]_I_ changes, expressed as F340/380, in control neurons and neurons exposed to FTY720 upon application of 4 μM s-Aβ. Corresponding quantification of s-Aβ-induced [Ca^2+^]_I_ responses is shown in (**C**) (Student’s t-test, P ≤ 0.001). (**D**) Representative temporal plots of [Ca^2+^]_I_ changes in control neurons and neurons exposed to FTY720 upon bath application of 50 μM NMDA. (**E**) Quantitative analysis of peak [Ca^2+^]_I_ responses evoked by NMDA in control neurons, and neurons pre-incubated with FTY720 in the presence or not of S1P-R1 and S1P-R3 antagonists (Kruskal–Wallis ANOVA, P < 0.001; Dunn’s test for comparison among groups, P < 0.05). (**F**) Time course analysis of FTY720 action on calcium influx through NMDARs. (**G**) Representative time plot analysis of [Ca^2+^]_I_ responses mediated by synaptic and extrasynaptic NMDARs, after MK801 block, in control neurons and neurons pre-incubated with FTY720 for 1 h. Corresponding quantification of synaptic and extrasynaptic [Ca^2+^]_I_ responses are shown in (**H**) (Mann-Whitney Rank Sum Test, P < 0.001) and (**I**) (Kruskal–Wallis ANOVA, P < 0.001; Dunn’s test for comparison among groups, P < 0.05).

Previous evidence indicates that s-Aβ forms activate acute calcium influx through NMDARs^7^,^32^,^60^, likely contributing to early dysregulation of calcium homeostasis (Fig. 1A). We thus investigated whether FTY720 may alter neuronal calcium responsiveness to acute challenge with s-Aβ or NMDA. We analysed calcium transients evoked by bath application of 4 μM s-Aβ (in Mg^2+^ free solution, 1 μM TTX and 20 μM CNQX to block AMPA/kainate receptors) in 12–15 DIV neurons, pre-incubated with FTY720 (200 nM) or vehicle for 1 h, during fura-2 loading. A significant reduction of calcium responses to s-Aβ was observed in FTY720-treated cultures (Fig. 3A–C). A similar decrease in calcium responses to 50 μM NMDA (in Mg^2+^ free solution, 1 μM TTX and 20 μM CNQX) was observed in neurons pre-treated with FTY720 for 1 h (Fig. 3D,E), but not upon shorter drug treatment (Fig. 3F). The modulation of NMDA-mediated calcium responses was completely prevented in the presence of the S1P-R1 and S1P-R3 selective antagonists W146 and CAY1044, indicating a S1P-R-dependent action of the drug. No change of peak calcium responses to 50 μM AMPA was observed in FTY720-treated neurons, suggesting specific modulation of NMDARs (AMPAR-mediated peak calcium transients expressed as DF340/380 -normalized values-: control = 1 ± 0.04, FTY720 = 1.056 ± 0.034, S1P-R1,3 antagonists = 1.05 ± 0.04; N = 3; One way Anova analysis of variance P = 0.398; Data not shown).

We next assessed the action of FTY720 on synaptic versus extrasynaptic NMDARs by employing a protocol to activate synaptic NMDARs and then isolate extrasynaptic NMDARs using the irreversible (in our experimental time-frame) NMDAR channel blocker MK801 (adapted from ref. 61). Briefly, neurons were exposed to 50 μM bicuculline in low Mg^2+^, 1 mM AP4, 20 μM CNQX and 30 μM D-serine, a selective co-agonist of synaptic NMDARs^9^, to enhance spontaneous synaptic transmission and opening of synaptic NMDARs, followed by MK801 application (10 μM), to block synaptic NMDARs. The extrasynaptic pools of NMDARs was then activated with 50 μM NMDA in Mg^2+^ free solution, 1 μM TTX and 20 μM CNQX (Fig. 3G–I). Lower calcium responses mediated by extrasynaptic NMDARs were detected in neurons treated with FTY720 (Fig. 3G and I), in line with the general decrease in NMDAR-mediated calcium responses induced by the drug (Fig. 3E). Importantly, FTY720 sensitivity of extrasynaptic responses was greater than the sensitivity of calcium rises mediated by total NMDARs (Fig. 3I versus E; Mann-Whitney Rank Sum Test P = 0.003), suggesting that the drug may inhibit mostly extrasynaptic NMDARs. Accordingly, analysis of peak calcium responses mediated by synaptic NMDARs upon bicuculline application showed no decrease in calcium influx under treatment with FTY720 (Fig. 3G,H). Calcium responses mediated by synaptic NMDARs were actually larger in the presence of the drug, suggesting an increase in synaptic NMDAR function (Fig. 3H).

### FTY720 decreases surface GLUN2B at extrasynaptic sites while increases surface GLUN2A and GLUN2B at synapses

**Figure 4:**
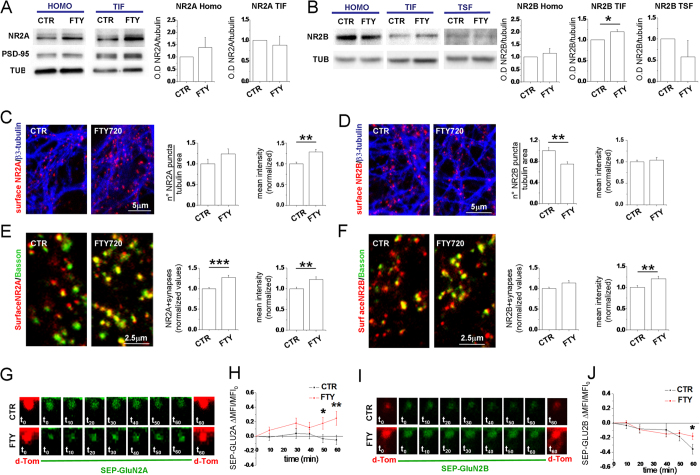
FTY720 modulates GLUN2B localization in the postsynaptic fraction and increases surface GLUN2B and GLUN2A expression at synapses. (**A**,**B**) Western blotting of the homogenate (HOMO), postsynaptic triton insoluble fraction (TIF) and triton soluble fraction (TSF) obtained from control and FTY720-treated hippocampal neurons (DIV14). Tubulin is used as loading control, while PSD-95 as a marker of postsynaptic fraction. FTY720 leads to an increased GLUN2B localization in the TIF leaving the total amount of GLUN2B unaltered while doesn’t alter GLUN2A localization in TIF (Student’s t-test, P = 0.0275 N = 4). (**C**) Confocal images of neurons live stained for GLUN2A, fixed and counterstained against β3-tubulin. Relative quantification of density of GLUN2A puncta are shown on the left (Mann-Whitney Rank Sum Test, P = 0.070) and mean intensity of GLUN2A clusters on the right (Mann-Whitney Rank Sum Test, P = 0.001). (**D**) Images of neurons live stained for GLUN2B and quantification of GLUN2B puncta density (Mann-Whitney Rank Sum Test, P = 0.003) and mean intensity (Student’s t-test, P = 0.610). (**E**,**F**) Images of neurons live stained for GLUN2A (**E**) or GLUN2B (**F**), fixed and counterstained against the presynaptic marker bassoon. Right histograms show quantification of GLUN2A positive synapses (Mann-Whitney Rank Sum Test, P ≤ 0.001) and GLUN2A puncta mean intensity (Mann-Whitney Rank Sum Test, P = 0.005) (number of analyzed puncta: control = 1010, FTY720 = 720; number of analyzed fields: control = 35; FTY720 = 30) or GLUN2B positive synapses (Mann-Whitney Rank Sum Test, P = 0.005) and GLUN2B puncta mean intensity (number of analyzed puncta: control = 1250, FTY720 = 807; number of analyzed fields: control = 37; FTY720 = 32). (**G**) Representative images of spines co-transfected with SEP-GluN2A(green) and dTom (red) acquired at the indicated time point in control and FTY720-treated neurons. (**H**) XY graph representing the DMFI/MFI_0_ of SEP-GluN2A over time (number of analysed spines: control = 40, FTY720 = 47; Mann-Whitney Rank Sum Test, P = 0.037 at t_50_ and P = 0.009 at t_60_). (**I**) Images of spines co-transfected with SEP-GluN2B and dTom as in G. (**J**) XY graph representing the DMFI/MFI_0_ of SEP-GluN2B over time (number of analysed spines: control = 15, FTY720 = 22; Mann-Whitney Rank Sum Test, P = 0.030 at t_60_).

To probe the molecular mechanisms underlying changes of functional NMDARs at extrasynaptic and synaptic sites we first investigated the effect of FTY720 on the expression and synaptic localization of GluN2A and GluN2B subunits. Hippocampal neurons were incubated in the absence or presence of FTY720 (200 nM, 1 h), and then GluN2A and GluN2B synaptic levels were analyzed. The Triton-Insoluble Fraction(TIF), which is enriched in PSD proteins^62^, was obtained from control and FTY720-treated neurons, and protein levels measured by Western blotting. The analysis revealed no changes in the expression of either subunits in the total homogenate (Fig. 4A,B). FTY720 treatment significantly increased GluN2B synaptic levels without affecting GluN2A immunostaining in the TIF. We measured the GluN2B levels in the triton-soluble extrasynaptic membrane fractions (TSF) and we detected a decrease, albeit not significant, in such fraction, suggesting a mobilization of GluN2B from the extrasynaptic to the synaptic sites. No detectable levels of GluN2A were observed in triton-soluble extrasynaptic membrane fractions (not shown), consistent with a dominant synaptic localization of the subunit, which remained stable in the synapse upon FTY720 exposure (Fig. 4A).

To further characterize changes in NMDARs distribution, we stained FTY720-treated and control hippocampal cultures with antibodies directed against extracellular epitopes of GluN2A and GluN2B. The mean intensity of GluN2A puncta increased significantly in FTY720-treated neurons, indicating that FTY720 promotes GluN2A membrane insertion (Fig. 4C, left). An increase in the density of surface GluN2A puncta was also evident, but the change was not statistically significant (Fig. 4C, right). By contrast, the density of surface GLUN2B puncta (Fig. 4D right panel) decreased significantly in FTY720-treated cultures. Of note, mean intensity of GLUN2B puncta persisting at the neuron surface after drug treatment was unaffected (Fig. 4D right panel), suggesting that FTY720 may cause internalization of a subset of GLUN2B-containing NMDARs.

Selective loss of surface GLUN2B-containing NMDARs at extrasynaptic sites may account for reduced Ca^2+^ influx through extrasynaptic NMDARs. We indirectly tested this hypothesis by quantifying synaptic GLUN2B puncta, co-localizing with the presynaptic marker bassoon. No decrease in the fraction of synapses expressing surface GLUN2B was observed upon FTY720 treatment (Fig. 4F left panel), suggesting that loss of surface GLUN2B receptors selectively occurs at extrasynaptic sites. Interestingly, mean intensity of GLUN2B synaptic clusters was significantly higher in FTY720-treated neurons (Fig. 4F right panel) as compared to control neurons, suggesting possible mobilization of GLUN2B-containing NMDARs towards the synaptic membrane and strengthening the data obtained by biochemical fractionation (Fig. 4A). In line with enhanced synaptic GluN2B staining, we observed a significant shift of GLUN2B from extrasynaptic to synaptic sites in FTY720-treated neurons (Fig. 4B). Thus, despite a global reduction of surface GluN2B, GluN2B expression increases on the synaptic membrane in neurons treated with FTY720.

Parallel analysis of surface GluN2A subunits at bassoon-positive synaptic sites showed an increased fraction of synapses expressing GluN2A subunits at the membrane (Fig. 4E left) and increased intensity of synaptic surface GluN2A fluorescence in FTY720-treated neurons (Fig. 4E right), indicating that FTY720 fosters GluN2A insertion in the synaptic membrane.

### FTY720 induces insertion into the synaptic membrane of new GLUN2A and mobilizes GLUN2B towards the synapse

We then monitored surface dynamics of GLUN2A and GLUN2B at postsynaptic spines upon FTY720 application by imaging 14 DIV neurons transfected with subunits tagged on the N terminus with a pH-sensitive form of GFP (SEP-GLUN2A; SEP-GLUN2B)^63^. SEP-tagged subunits are mostly non-fluorescent when trapped intracellularly and display fluorescence when on the cell surface. To demarcate spine morphology, neurons were co-transfected with td-Tomato. To determine whether FTY720 induces exocytosis of GLUN2A/GLUN2B on the surface of spines we measured SEP fluorescence (mean fluorescence intensity) in circular region of interest (ROIs) manually positioned within the spines before and up to 1 h after drug application. We observed a significant increase in SEP-GLUN2A signal in FTY720-treated spines compared to control spines at 50 and 60 min of recording (Fig. 4G and H). Similarly, after 60 min of recording, SEP-GLUN2B signal was significantly higher in spines treated with the drug compared to control spines, which showed a general tendency to decrease over time (Fig. 4I and J). The increase in surface expression of GLUN2A and GLUN2B at postsynaptic spines was associated to a shift towards a more mature (mushroom) spine morphology (Supplementary Fig. 2).

### FTY720 increases the amplitude of NMDAR-mEPSCs, without changing in the ratio of synaptic GLUN2A/GLUN2B subunit

**Figure 5:**
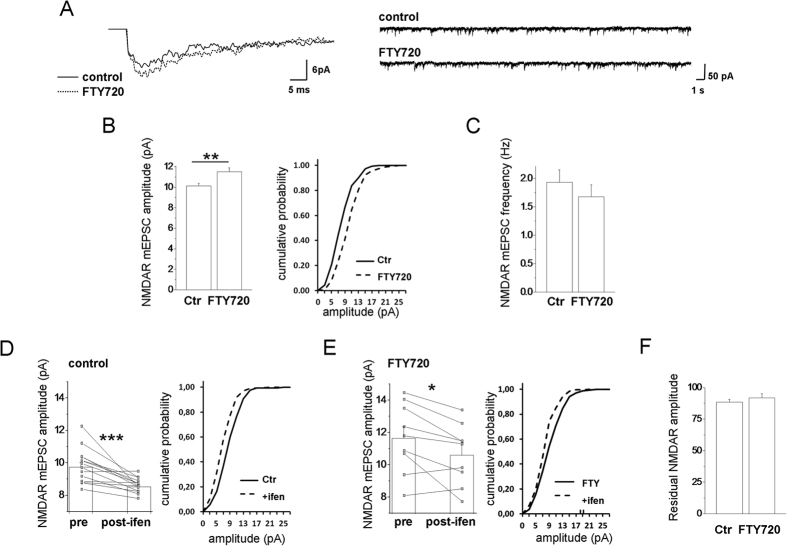
FTY720 increases the amplitude of NMDAR-mediated mEPSCs. (**A**) Mean NMDAR-mediated mEPSCs from control and FTY720-treated neurons (left) and corresponding representative traces (right). (**B**) Summary histogram showing the mean amplitudes of NMDAR-mediated mEPSCs from control and FTY720-treated neurons (number of cells: ctr = 35, FTY720 = 26; Student’s t-test, P = 0.002) (B-left); corresponding cumulative distributions of peak amplitudes from single representative cells (Kolmogorov-Smirnov test, P ≤ 0.001) (B-right). (**C**) Summary histogram showing mean frequencies of neurons in B-left (Mann-Whitney Rank Sum test P = 0.507). (**D**,**E**) Summary histograms and line series plots showing the block of ifenprodil on mEPSC amplitude in control (**D**) and FTY720-treated neurons (**E**) (number of cells: ctr = 14, FTY720 = 9; ctr basal vs ifenprodil: paired t-test, P = <0.001; FTY basal vs ifenprodil: paired t-test, P = 0.021) (D,E-left) and corresponding peak amplitude cumulative distributions from single representative cells (D,E-right) (ctr basal vs ifenprodil: Kolmogorov-Smirnov test, P ≤ 0.001; FTY basal vs ifenprodil: Kolmogorov-Smirnov test, P ≤ 0.001). (**F**) Residual NMDA mEPSC amplitude after Ifenprodil block (% residual amplitude ctr vs FTY: student t-test, P = 0.422).

We finally investigated how changes in synaptic NMDARs driven by FTY720 impact miniature NMDAR-excitatory postsynaptic currents (NMDAR-mEPSCs) induced by synaptically released glutamate. We recorded NMDAR-mEPSCs from 14–16 DIV hippocampal neurons at −60 mV pretreated or not with FTY720, in the presence of TTX, bicuculline, strychnine (1 μM), D-serine or glycine (10 μM) and CNQX to block detection of fast AMPAR-mediated mEPSCs component, and in Mg^2+^ -free medium to allow recording of the slow NMDAR component. We observed a significant increase in the amplitude of NMDAR-mEPSCs in FTY720-treated neurons as compared controls (Fig. 5A,B). The increase in NMDAR-mEPSC amplitude was not associated with frequency changes (Fig. 5C), consistent with a postsynaptic action of FTY720. Analysis of NMDAR-mEPSC sensitivity to the GLUN2B-selective antagonist ifenprodil (5 μM) showed a similar block of NMDAR-mEPSCs amplitude in control and FTY720-treated neurons (Fig. 5D–F). This is consistent with an increased content of functional GLUN2B- and GLUN2A-containing NMDARs and no alteration in the ratio of synaptic GLUN2A/GLUN2B subunit.

Collectively these data show that FTY720 favours functionality of synaptic GluN2A- and GLUN2B-containing NMDARs.

## Discussion

Our study strongly suggests a mechanistic link between neuroprotective effects of Fingolimod against Aβ–induced neurodegeneration and rapid relocation of functional NMDARs in cultured hippocampal neurons. Precisely, we show that the activation of S1P-R1 and S1P-R3 by Fingolimod drives quite rapid internalization of GLUN2B subunit at extrasynaptic regions and GLUN2B mobilization to the synapse, within a time scale of 1 h, as revealed by Western blotting in the synaptic and extrasynaptic membrane fractions and imaging studies in hippocampal cultures. Augmented GLUN2B at the synapse is accompanied by increased surface GLUN2B expression, as indicated by quantitative immunofluorescence analysis and live imaging with SEP-GLUN2B. Rapid mobilization of GLUN2B from extrasynaptic regions is consistent with the well-known mobility of the subunit, that is dynamically regulated by neuronal activity^64^ and by several secreted molecules, such as neurotrophins^65^. Overall reduction of surface GLUN2B induced by FTY720 suggests that increased GLUN2B expression on the synaptic membrane results from lateral movement of extrasynaptic GLUN2B clusters, persisting on the cell surface after treatment with the drug. However, we cannot exclude that new GLUN2B subunits could be inserted at synaptic sites following endocytosis from extrasynaptic regions upon drug treatment. Analysis of GLUN2B phosphorylation at residues controlling stabilization and anchoring of GLUN2B to neuron surface, such as Tyr1472^11^,^66^,^67^,^68^ will provide further insights into the molecular mechanism underling GLUN2B relocation to the synapse.

Aside from GLUN2B, FTY720 changes the subcellular distribution of GLUN2A-containing NMDARs, by driving rapid insertion of new GLUN2A subunits into the synaptic membrane. Immunofluorescence analysis of surface GLUN2A and the use of SEP-GLUN2A indicate a clear upregulation of the subunit on neuronal membrane, with no alteration in its location, which remains stable in the synaptic fractions.

As a result of GLUN2B mobilization to the synapse and GLUN2A insertion into the synaptic membrane, functional NMDARs raise at synaptic regions while decrease extrasynaptically. This is evidenced by enhanced NMDAR-mEPSC amplitude with no changes in the ratio of synaptic GLUN2A/GLUN2B subunit and by decreased calcium influx through extrasynaptic NMDARs in neurons treated with Fingolimod. Thus, FTY720 emerges from the current study as key regulator of NMDAR trafficking, able to rapidly change the ratio of synaptic versus extrasynaptic functional NMDARs. This mechanism may be behind augmented s-Aβ binding to synapses in FTY720-treated neurons.

Long standing evidence indicates that differentially located NMDARs are coupled to different intracellular cascades, with synaptic NMDARs being coupled to pro-survival pathways, primarily through nuclear Ca^2+^ signaling, while extracellular NMDARs -present on the cell body or the dendritic shaft- activating several pro-death cascades^69^. While unbalance between synaptic and extrasynaptic NMDAR activity has been indicated as a common feature of neurological disorders, enhancement of synaptic NMDARs and disruption of extrasynaptic NMDAR-dependent death pathways have been proposed as therapeutic strategy for brain diseases^12^. Here we strongly suggest that FTY720 renders neurons resistant to Aβ toxicity exactly through this mechanism, favouring synaptic versus extrasynaptic NMDAR function through S1P-R activation. Indeed, the increased ratio of synaptic/extrasynaptic NMDARs driven by FTY720 negatively correlates with the amplitude of harmful calcium responses of neurons to s-Aβ. Furthermore, it negatively correlates with early alterations of basal calcium concentration, which are detectable in neurons 1 h after treatment with s-Aβ. Conversely, pharmacological block of S1P-R1 and S1P-R3, which prevents the loss of functional extrasynaptic NMDARs induced by Fingolimod (and by S1P), completely neutralizes the positive action of the drug on calcium homeostasis. Of note, calcium responses mediated by extrasynaptic NMDARs become even larger in the presence of S1P-R1 and S1P-R3 antagonists. This evidence suggests that endogenous S1P may tonically inhibit extrasynaptic NMDARs, thereby constitutively limiting glutamate excitotoxicity. To our knowledge these data represent first evidence that pharmacological modulation of S1P-Rs differentially regulates synaptic and extrasynaptic NMDARs and offers a realistic approach to interfere with early events in the excitotoxic cascade in neurodegenerative diseases.

By identifying NMDARs as key downstream effectors of S1P-Rs, responsible for early FTY720 neuroprotection, our data support and complement recent studies showing that FTY720 makes neurons resistant to excitotoxic concentration of NMDA^30^,^40^,^52^ and attenuates s-Aβ toxicity^31^. According to these studies, prolonged treatment with FTY720 (for at least 24 h *in vitro*) enhances the expression of BDNF, a neurotrophin involved in synaptic plasticity, downstream of S1P-R activation and ERK1/2 signalling, and restores normal BDNF expression in a mouse model of Rett syndrome^40^ and in mice injected with neurotoxic Aβ^44^. It should be stressed, however, that the very early protective action of FTY720 we uncovered in our study, is not consistent with timing of induction of BDNF transcript. Although applied in the non phosphorylated, inactive form, FTY720 relocates GLUN2B and changes the ratio of synaptic/extrasynaptic NMDARs in only 1 h *in vitro*. Within this time window, the drug also shifts Aβ binding to synaptic regions and significantly reduces damage to neurons when co-applied with toxic Aβ species. On the other hand, a rapid increase in BDNF protein translation has been recently described upon acute dose of the NMDAR antagonist ketamine^70^,^71^. Thus, we cannot exclude that de-repression of BDNF translation may occur and contribute to fast protective signaling downstream inhibition of extrasynaptic NMDARs upon FTY720 treatment. Further work needs to be done to explore possible changes of BDNF protein upon acute FTY720 treatment.

It has been recently shown that FTY720 is equally efficient as the NMDAR antagonist memantine, an approved drug to treat AD symptoms, in partly reversing changes of gene expression induced by Aβ injection in rodents^44^. By showing that FTY720 acts as functional antagonist of extrasynaptic GLUN2B-containing NMDARs, we here provide a possible mechanism underlying the positive action of the drug on the expression of genes altered by Aβ.

More importantly, synaptic relocation of GLUN2B-containing NMDARs mediated by FTY720 might have direct impact on synaptic plasticity and memory processes and underlie the increase in cognitive performance observed in AD mice after drug treatment (this study Supplementary Fig. 1; Asle-Rousta, 2013 #696}^43^,^44^. Consistent with this possibility recent works show that FTY720 significantly facilitates both LTP in hippocampal slices^24^ and that synaptic activation of GLUN2B-containing NMDARs is essential for the formation of LTP, the physiological mechanism behind memory^72^,^73^.

Thus, beyond a fast defensive response against s-Aβ excitoxicity, facilitation of synaptic versus extrasynaptic NMDAR function makes this drug, acting on multiple brain cell targets, a really promising neuroprotective agent, able to directly influence the key postsynaptic effectors of synaptic plasticity, i.e. NMDARs, in a S1P-Rs-dependent manner.

## Materials and Methods

### Animals

For behavioral analysis 12-month-old male and female APPswe/PS1dE9 transgenic mice and age-matched littermates were used. All the experimental procedures followed the guidelines established by the Italian Council on Animal Care (L.D. no 26/2014) and were approved by the Milano University Bioethical Committee. All efforts were made to minimize the number of subjects used and their suffering. APPswe/PS1dE9 mice and their littermates were housed in independent cages, with free access to food and water at 22 °C and with a 12-h alternating light/dark cycle. For behavioral profile animals were tested once for each test. All the tests were carried out between 8.00 and 14.00.

### Motor function

Spontaneous motor activity was evaluated in an automated activity cage placed in a sound attenuating room as previously described^74^. Cumulative horizontal beam breaks were counted for 15 min.

### Passive avoidance

The apparatus consisted of two compartments, one light and one dark, connected via a sliding door. In the acquisition trial, each mouse was placed in the light compartment and allowed to enter the dark compartment as previously described^75^. The time(in s) taken to do so was recorded. Once the mouse was in the dark compartment, the sliding door was closed and an unavoidable electric shock (1 mA for 1 s) delivered via the paws. The animal was then placed back in the home cage until the retention trial. The retention trial was carried out 24 h after the acquisition trial, by positioning the mouse in the light compartment and recording the time taken to enter the dark compartment (retention latency). An increased retention latency indicates that the animal has learned the association between the shock and the dark compartment. During the retention trial, a cut-off time of 180 s was used. An experimenter blind to the treatment group manually recorded the latency time.

### Novel object recognition (NOR)

The test was conducted over a two-day period in an open plastic arena (60 cm × 50 cm × 30 cm), as previously described^74^. Animals were habituated to the test arena for 10 min on the first day. After 1-day habituation, mice were subjected to familiarization (*T*_1_) and novel object recognition (*T*_2_). During the initial familiarization stage, two identical objects were placed in the center of the arena equidistant from the walls and from each other. Each mouse was placed in the center of the arena between the two objects for a maximum of 10 min or until it had completed 30 s of cumulative object exploration. Object recognition was scored when the animal was within 0.5 cm of an object with its nose toward the object. Exploration was not scored if a mouse reared above the object with its nose in the air or climbed on an object. Mice were returned to the home cage after familiarization and then tested again after a delay of 2 hours. A novel object (never seen before) took the place of one of the two familiars. An experimenter blind to the treatment group manually recorded the exploration times to the objects for each animal. Performance was evaluated by discrimination index (N − F/N + F) where N = time spent exploring the new object during *T*_2_, F = time spent exploring the familiar object during *T*_2_.

### Nest building

The nest building test was performed in the home cage led with 0.5 cm bedding according to ref. 76. Each cage was supplied with a “Nestlet”, a 5 cm square of pressed cotton batting, one hour before the dark phase. Results were assessed the next morning. The nests were assessed on a 5-point scale:

1. The Nestlet is largely untouched (>90% intact).
2. The Nestlet is partially torn up (50–90% remaining intact).
3. The Nestlet is mostly shredded but often there is no identifiable nest site: <50% of the Nestlet remains intact but <90% is within a quarter of the cage floor area, i.e. the cotton is not gathered into a nest but spread around the cage. Note: the material may sometimes be in a broadly defined nest area but the critical definition is that 50–90% has been shredded.
4. An identifiable, but flat nest: >90% of the Nestlet is torn up, the material is gathered into a nest within a quarter of the cage floor area, but the nest is flat, with walls higher than mouse body height (curled up on its side) on less than 50% of its circumference.
5. A (near) perfect nest: >90% of the Nestlet is torn up, the nest is a crater, with walls higher than mouse body height on more than 50% of its circumference.

This procedure was repeated each day for 4 consequent days. An experimenter blind to the treatment group manually evaluated the nest building with an appropriate score.

### Hippocampal neurons

Primary neuronal cultures were obtained from the hippocampi of 18-day-old fetal Sprague Dawley rats of either sex (Charles River Italia), or C57BL/6 GFP transgenic mice with the GFP gene controlled by the actin promoter^77^ (of either sex). Briefly, dissociated cells were plated onto poly-L-lysine (Sigma Aldrich, St. Louis, MO) treated coverslips and maintained in Neurobasal with 2% B27 supplement (Invitrogen, Carlsbad, CA), antibiotics, glutamine and glutamate. Neurons were used at 9–16 DIV.

### Aβ 1–42 preparation

To prepare soluble Aβ 1–42, the peptide (Anaspec, Fremont, CA) was initially monomerized by dissolving it in 100% hexafluoroisopropanol (Sigma, St. Louis, MO, USA) to obtain a 1 mM solution and then aliquoted in sterile microcentrifuge tubes. The hexafluoroisopropanol was removed under vacuum using a SpeedVac and the peptide film was stored (desiccated) at −80 °C. Soluble Aβ was prepared as previously described^78^. Briefly, the peptide film was freshly resuspended in 100% DMSO to 5 mM, further diluted to 100 μM in F-12 medium (Invitrogen, Paisley PA4 9RF, UK) and incubated for 24 h at 4 °C. Following incubation it was centrifuged at 14,000 g for 10 min at 4 °C and the soluble forms were collected in the supernatant.

For SPR and ThT studies depsi-Aβ 1–42 was synthesized in-house, as previously^79^. The depsi-peptide is much more soluble than the native one and has much less propensity to aggregate, preventing the spontaneous formation of seeds in solution. Aβ 1–42 was then obtained from the depsi-peptide by a “switching” procedure involving a change in pH^80^. The solution was diluted in 10 mM PBS, pH 7.4, to a final concentration of 100 μM and incubated at 25 °C in quiescent conditions for different times. We used freshly prepared solutions (t = 0) to have Aβ monomers only, Aβ solutions incubated for 5 h (t = 5 h) for maximal Aβ oligomer enrichment, whereas incubation for 24 h allowed formation of protofibrils^59^. To prepare Aβ 1–42 fibrils, the switched peptide solution was diluted with water to 100 μM, acidified to pH 2.0 with 1 M HCl, and left to incubate from 20 to 24 h at 37 °C.

### SPR studies

SPR studies were carried out with the ProteOn XPR36 Protein Interaction Array System (Bio-Rad Laboratories, Hercules, CA) based on SPR technology. Aβ 1–42 species were immobilized in parallel-flow channels of the same GLC sensor chip (Biorad) using amine-coupling chemistry, as previously described^58^. A reference surface was always prepared in parallel using the same immobilization procedure but without addition of the peptide. The potential interaction of FTY720 was then evaluated by injecting four different concentrations of the compound (125–1000 nM), as well as the vehicle over the immobilized ligands or control surfaces, in parallel, at the same time. The signal in the surfaces immobilizing Aβ was corrected by subtracting the nonspecific response observed in the reference surface. In another set of SPR experiments, the anti-Aβ antibody 4G8 (Covance, Princeton, NJ, USA) was immobilized on GLC sensor chips (Bio-Rad) using amine-coupling chemistry, as previously described^59^. A reference surface, using the same immobilization procedure but without addition of the antibody, was always prepared in parallel. Oligomer-enriched Aβ1–42 solutions (i.e. incubated for 5 h) diluted to a final Aβ1–42 concentration of 1 μM, were flowed over the chip surfaces for 2 min, followed by 10-min flow with running buffer (dissociation phase). According to our previous data, the SPR signal measured at the end of the session is only due to oligomers (Aβ monomers completely dissociate from 4G8 after 10 min in running buffer, whereas most oligomers remain pseudo-irreversibly bound to the antibody ref. 59). In order to evaluate the effect of FTY720 on the oligomers formation, Aβ1–42 solutions were incubated for 5 h in the absence or presence of FTY720 and then injected over immobilized 4G8. The running buffer, also used to dilute the samples, was 10 mM PBS containing 150 mM NaCl and 0.005% Tween 20 (PBST). All these assays were performed at 25 °C. The sensograms (time-course of the SPR signal in RU) were normalized to a baseline value of 0. The signal observed on the surfaces immobilizing antibodies was corrected by subtracting the nonspecific response observed on the reference surface.

### ThT studies

A freshly prepared solution of Aβ 1–42 (2 μM) was incubated with 10 μM thioflavinT (ThT) with or without FTY720, and the ThT fluorescence was monitored for 13 h^79^. In‐situ ThT kinetic experiments were done on a plate reader (M200 Infinity, Tecan) using a 96‐well black plate with flat transparent bottom. The dye was excited at 440 nm and emission was taken at 495 nm.

### Calcium Imaging

9–14 DIV hippocampal neurons were loaded with 2 μM Fura-2 pentacetoxy methylester for 40 min at 37 °C, washed and transferred to the recording chamber of an inverted microscope (Axiovert 100; Zeiss, Oberkochen, Germany) equipped with a calcium imaging unit Polychrome V (TILL Photonics, Germany) as described^81^. Images were collected with a CCD Imago-QE camera (TILL Photonics GmbH) and analyzed with TILLvisION 4.5.66 software. After excitation at 340 and 380 nm wavelengths, the emitted light was acquired at 505 nm at 1 Hz. Calcium concentration was expressed as F340/F380 fluorescence ratio. The ratio values in selected region of interest corresponding to neuronal cell bodies were calculated from sequences of images to obtain temporal analysis. Basal calcium concentration was recorded from at least 100 neurons/condition in each experiment.

### Activation of synaptic/extrasynaptic NMDARs

Neurons were exposed to low Mg^2+^ KRH (125 mM NaCl, 5 mM KCl, 1.2 mM KH_2_PO, 2 mM CaCl_2_, 0.6 mM MgSO_4_, 6 mM D-glucose, and 25 mM HEPES/NaOH, pH 7.4) containing 50 μM bicuculline, 1 mM APV, 30 μM D-serine and 20 μM CNQX to activate synaptic NMDARs followed by 10 μM MK801 for 3 min to block synaptic NMDARs. Then neurons were washed several times to remove unbound MK801, followed by incubation in Mg^2+^ free KRH containing CNQX and TTX and 50 μM NMDA to selectively activate extrasynaptic NMDARs.

### Surface staining for GLUN2A and GLUN2B. Quantification of total surface and synaptic signal

Living neurons were incubated for 10 min with rabbit antibodies directed against extracellular epitopes of GLUN2A (1:100, Invitrogen, CA, USA) or GLUN2B (1:100, Alomone Labs, Jerusalem, Israel), washed and fixed with 4% paraformaldehyde and 4% sucrose. The following antibodies were used to double stain the cultures: guinea pig anti-Bassoon (1:500, Synaptic System, Goettingen, Germany), mouse anti-beta III tubulin (1:500; Promega Corporation, Madison, WI, USA). Secondary antibodies were conjugated with Alexa-488, Alexa-555, Alexa-633 (Alexa-Invitrogen, San Diego, CA). Images were acquired using a Leica SPE confocal microscope equipped with an ACS APO × 63/1.30 oil objective (Leica Microsystems, Solms, Germany. Acquisition parameters (i.e., laser power, gain and offset) were kept constant among different experimental settings. Surface GLUN2A/GLUN2B staining was quantified as follows. Number of GLUN2A/GLUN2B puncta were measured using Image J 1.46r software in GLUN2A/GLUN2B fluorescence images after setting a fixed threshold using the ‘analyze particle’ function. Number of GLUN2A/GLUN2B puncta in each field (devoid of neuronal cell bodies) were then normalized to β-tubulin area after setting a fixed threshold to obtain density of GLUN2A/GLUN2B puncta. All data are results of at least 3 independent experiments. GLUN2A/GLUN2B puncta colocalizing with bassoon were manually selected to measure mean intensity and size of single puncta. Approximately 75 spines per field were measured in at least ten fields per independent experiments.

### Analysis of s-Aβ binding to neurons

s-Aβ was prepared as described above and incubated for 1 h with mouse neurons from C57BL/6J-GFP transgenic mice or wild type mouse/rat hippocampal neurons. Neurons were then fixed and stained with rabbit anti-Shank-2 (1:500, NeuroMab, Davis, CA). s-Aβ binding to eGFP positive neuritis was quantified as follows. Aβ and eGFP double-positive puncta (bound Aβ) were revealed by generating a Aβ/eGFP double-positive image using the ‘and’ option of ‘image calculator’ function. A fixed threshold was then set in the double-positive image and number of double-positive puncta was quantified using the ‘analyze particle’ function. Total eGFP fluorescence area was directly measured in the green channel, after setting a fixed threshold. Finally, number of Aβ/eGFP colocalizing puncta was normalized to eGFP area in each field to obtain density of bound Aβ puncta. A similar procedure was used to quantify s-Aβ binding to postsynaptic spines. Aβ and Shank-2 double-positive puncta (synaptic Aβ) were revealed by generating a Aβ/Shank-2 double-positive image. A fixed threshold was then set in the double-positive image and number of Aβ-positive puncta was measured and normalized to total number of Shank-2 puncta to obtain the fraction of Aβ positive postsynaptic spines.

### Live imaging with SEP constructs

Control and FTY720-treated neurons were maintained in neurobasal medium without phenol red, supplemented with B27 in the incubator chamber of an inverted LSM510 confocal microscope (Zeiss, Jena, Germany). SEP-GluN2A/dTom or SEP-GluN2B/dTom images were acquired, every 10 min for 1 h. Mean fluorescence intensity (MFI) was measured using ImageJ software at ROIs selected within only those spines that were stable over 1 h, after setting a fixed threshold. ΔMFI/MFI_0_, was then calculated and plotted.

### Electrophysiological recordings

Whole-cell voltage clamp recordings were performed using a MultiClamp 700A amplifier (Axon Instruments) coupled to a pCLAMP 10 Software (Molecular Devices), and using an inverted Axiovert 200 microscope (Zeiss). NMDA-mEPSCs were recorded from 14–15 DIV hippocampal neurons plated at a density of 1.7 × 10^5^ neurons/coverslip at room temperature (20–25 °C). Holding potential was set at −60 mV and a Cesium Gluconate (CsGluc) internal solution was used: 130 mM CsGluc, 8 mM CsCl, 2 mM NaCl, 10 mM HEPES, 4 mM EGTA, 4 mM MgATP, 0.3 mM Tris-GTP (pH 7.3, adjusted with CsOH). Mg^2+^ -free KRH was used as external solution and recordings were performed in the presence of 1 μM tetrodotoxin, 20 μM CNQX, 20 μM bicuculline, all from Tocris (Bristol, UK), 1 μM strychnine, 10 μM D-serine or 10 μM glycine, all from Sigma Aldrich (St. Louis, MO). Signals were sampled at 10 kHz and filtered to 2 kHz. Recording pipettes were fabricated from capillary glass using a two-stage puller (Narishige, Japan) to have a tip resistances of 3–5 MΩ. Series resistance was monitored before and during experiments. In a set of experiments we applied 100 μM APV to determine whether slow currents recorded were actually mediated by NMDAR. NMDA-mEPSC traces were analyzed with Clampfit Software using a threshold of 3 pA. FTY720 was administrated at 200 nM for 1 h.

### TIF preparation

Triton insoluble fractions (TIFs) were isolated from DIV14 hippocampal neurons as previously described^82^. Cells were homogenized in ice-cold sucrose 0.32 M containing 1 mM Hepes, 1 mM MgCl_2_, 1 mM EDTA, 1 mM NaHCO_3_, 0.1 mM PMSF, at pH 7.4 and centrifuged at 800 × *g* for 5 min. The resulting supernatant (S1) was centrifuged at 13,000 × *g* for 15 min to obtain a crude membrane fraction (P2 fraction). The pellet was resuspended in buffer containing 75 mM KCl and 1% Triton X-100 and centrifuged at 100,000 × *g* for 1 h at 4 °C. The supernatant was stored and referred as Triton soluble fraction (TSF). The final pellet (TIF) was homogenized in a glass-glass potter in 20 mM Hepes and stored at −80 °C until processing. All purifications were performed in presence of a complete set of protease inhibitors (Complete, Roche) and of both Ser/Thr- and Tyr-phosphatase inhibitor cocktails (Sigma-Aldrich).

### Reagents

FTY720 and CAY10444 were from Cayman Chemicals (Ann Arbor, MI) and W146 from Avanti Polar Lipids (Alabaster, Alabama). Tetrodotoxin, CNQX, bicuculline, APV, NMDA, AMPA were all from Tocris (Bristol, UK). Strychnine, D-serine, glycine, Fura-2/am, MK801, from Sigma Aldrich (St. Louis, MO).

### Data analysis

All data are expressed as means ± SEM. Behavioral data were statistically analyzed and displayed by Prism 6 software (GraphPad, San Diego, CA). Two-way ANOVA for multiple comparisons, followed by Bonferroni’s test was used to evaluate the difference among groups for behavioural data. Kolmogorov-Smirnov test was used for comparing cumulative distributions in NMDA mEPSC amplitude analysis; the test was performed using Origin8 software. All other data were first tested for normal distribution with SigmaStat 3.5 software, and the appropriate statistical test was been used (see figure legends). The accepted level of significance was p ≤ 0.05, indicated by an asterisk; those at P was ≤0.01 are indicated by double asterisks, while the ones at P ≤ 0.001 are indicated by triple asterisk.

Sample size was chosen according to G*Power software. Not healthy neurons were excluded from the analysis, based on basal calcium levels, morphology and electrophysiological parameters (resting membrane potentials, series resistance, leak currents), which were monitored at the beginning and during recordings. We chose comparable samples (neuronal coverslips from the same preparation, plated at the same cell density/mice) and utilized them randomly for controls and experimental treatments.

## Additional Information

**How to cite this article:** Joshi, P. *et al*. Fingolimod Limits Acute Aβ Neurotoxicity and Promotes Synaptic Versus Extrasynaptic NMDA Receptor Functionality In Hippocampal Neurons. *Sci. Rep.* **7**, 41734; doi: 10.1038/srep41734 (2017).

**Publisher's note:** Springer Nature remains neutral with regard to jurisdictional claims in published maps and institutional affiliations.

## Supplementary Material

Supplementary Material

## References

[b1] De Felice F. G. . Abeta oligomers induce neuronal oxidative stress through an N-methyl-D-aspartate receptor-dependent mechanism that is blocked by the Alzheimer drug memantine. J. Biol. Chem. 282, 11590–11601, doi: 10.1074/jbc.M607483200 (2007). 17308309

[b2] Decker H., Lo K. Y., Unger S. M., Ferreira S. T. & Silverman M. A. Amyloid-beta peptide oligomers disrupt axonal transport through an NMDA receptor-dependent mechanism that is mediated by glycogen synthase kinase 3beta in primary cultured hippocampal neurons. J. Neurosci. 30, 9166–9171, doi: 10.1523/JNEUROSCI.1074-10.2010 (2010). 20610750 PMC6632489

[b3] Mizoguchi H. . Matrix metalloprotease-9 inhibition improves amyloid beta-mediated cognitive impairment and neurotoxicity in mice. J. Pharmacol. Exp. Ther. 331, 14–22, doi: 10.1124/jpet.109.154724 (2009). 19587312

[b4] Deshpande A., Mina E., Glabe C. & Busciglio J. Different conformations of amyloid beta induce neurotoxicity by distinct mechanisms in human cortical neurons. J. Neurosci. 26, 6011–6018, doi: 10.1523/JNEUROSCI.1189-06.2006 (2006). 16738244 PMC6675207

[b5] Gomez-Ramos A., Diaz-Hernandez M., Cuadros R., Hernandez F. & Avila J. Extracellular tau is toxic to neuronal cells. FEBS Lett. 580, 4842–4850, doi: 10.1016/j.febslet.2006.07.078 (2006). 16914144

[b6] Lacor P. N. . Abeta oligomer-induced aberrations in synapse composition, shape, and density provide a molecular basis for loss of connectivity in Alzheimer’s disease. J. Neurosci. 27, 796–807, doi: 10.1523/JNEUROSCI.3501-06.2007 (2007). 17251419 PMC6672917

[b7] Texido L., Martin-Satue M., Alberdi E., Solsona C. & Matute C. Amyloid beta peptide oligomers directly activate NMDA receptors. Cell Calcium 49, 184–190, doi: 10.1016/j.ceca.2011.02.001 (2011). 21349580

[b8] Decker H. . N-methyl-D-aspartate receptors are required for synaptic targeting of Alzheimer’s toxic amyloid-beta peptide oligomers. J. Neuro. chem. 115, 1520–1529, doi: 10.1111/j.1471-4159.2010.07058.x (2010). 20950339

[b9] Papouin T. . Synaptic and extrasynaptic NMDA receptors are gated by different endogenous coagonists. Cell 150, 633–646, doi: 10.1016/j.cell.2012.06.029 (2012). 22863013

[b10] Leveille F. . Neuronal viability is controlled by a functional relation between synaptic and extrasynaptic NMDA receptors. Faseb J. 22, 4258–4271, doi: 10.1096/fj.08-107268 (2008). 18711223

[b11] Lau C. G. & Zukin R. S. NMDA receptor trafficking in synaptic plasticity and neuropsychiatric disorders. Nat. Rev. Neurosci. 8, 413–426, doi: 10.1038/nrn2153 (2007). 17514195

[b12] Hardingham G. E. & Bading H. Synaptic versus extrasynaptic NMDA receptor signalling: implications for neurodegenerative disorders. Nat. Rev. Neurosci. 11, 682–696, doi: 10.1038/nrn2911 (2010). 20842175 PMC2948541

[b13] Bordji K., Becerril-Ortega J., Nicole O. & Buisson A. Activation of extrasynaptic, but not synaptic, NMDA receptors modifies amyloid precursor protein expression pattern and increases amyloid-ss production. J. Neurosci 30, 15927–15942, doi: 10.1523/JNEUROSCI.3021-10.2010 (2010). 21106831 PMC6633754

[b14] Li S. . Soluble Abeta oligomers inhibit long-term potentiation through a mechanism involving excessive activation of extrasynaptic NR2B-containing NMDA receptors. J Neurosci 31, 6627–6638, doi: 10.1523/JNEUROSCI.0203-11.2011 (2011). 21543591 PMC3100898

[b15] Kervern M. . Selective impairment of some forms of synaptic plasticity by oligomeric amyloid-beta peptide in the mouse hippocampus: implication of extrasynaptic NMDA receptors. J Alzheimers Dis 32, 183–196, doi: 10.3233/JAD-2012-120394 (2012). 22785392

[b16] Tackenberg C. . NMDA receptor subunit composition determines beta-amyloid-induced neurodegeneration and synaptic loss. Cell Death Dis 4, e608, doi: 10.1038/cddis.2013.129 (2013). 23618906 PMC3641351

[b17] Kappos L. . A placebo-controlled trial of oral fingolimod in relapsing multiple sclerosis. N. Engl. J. Med. 362, 387–401, doi: 10.1056/NEJMoa0909494 (2010). 20089952

[b18] Cohen J. A. . Oral fingolimod or intramuscular interferon for relapsing multiple sclerosis. N Engl. J. Med. 362, 402–415, doi: 10.1056/NEJMoa0907839 (2010). 20089954

[b19] Brinkmann V. . The immune modulator FTY720 targets sphingosine 1-phosphate receptors. J. Biol. Chem. 277, 21453–21457, doi: 10.1074/jbc.C200176200 (2002). 11967257

[b20] Mandala S. . Alteration of lymphocyte trafficking by sphingosine-1-phosphate receptor agonists. Science 296, 346–349, doi: 10.1126/science.1070238 (2002). 11923495

[b21] Mullershausen F. . Persistent signaling induced by FTY720-phosphate is mediated by internalized S1P1 receptors. Nat. Chem. Biol. 5, 428–434, doi: 10.1038/nchembio.173 (2009). 19430484

[b22] Kataoka H. . FTY720, sphingosine 1-phosphate receptor modulator, ameliorates experimental autoimmune encephalomyelitis by inhibition of T cell infiltration. Cell Mol. Immunol. 2, 439–448 (2005). 16426494

[b23] Foster C. A. . Brain penetration of the oral immunomodulatory drug FTY720 and its phosphorylation in the central nervous system during experimental autoimmune encephalomyelitis: consequences for mode of action in multiple sclerosis. J Pharmacol Exp Ther 323, 469–475, doi: 10.1124/jpet.107.127183 (2007). 17682127

[b24] Hait N. C. . Active, phosphorylated fingolimod inhibits histone deacetylases and facilitates fear extinction memory. Nat Neurosci 17, 971–980, doi: 10.1038/nn.3728 (2014). 24859201 PMC4256678

[b25] Briard E. . BZM055, an iodinated radiotracer candidate for PET and SPECT imaging of myelin and FTY720 brain distribution. Chem. Med. Chem. 6, 667–677, doi: 10.1002/cmdc.201000477 (2011). 21280229

[b26] Miron V. E. . Fingolimod (FTY720) enhances remyelination following demyelination of organotypic cerebellar slices. Am. J. Pathol. 176, 2682–2694, doi: 10.2353/ajpath.2010.091234 (2010). 20413685 PMC2877831

[b27] Choi J. W. . FTY720 (fingolimod) efficacy in an animal model of multiple sclerosis requires astrocyte sphingosine 1-phosphate receptor 1 (S1P1) modulation. Proc. Natl. Acad. Sci. USA 108, 751–756, doi: 10.1073/pnas.1014154108 (2011). 21177428 PMC3021041

[b28] Verderio C. . Myeloid microvesicles are a marker and therapeutic target for neuroinflammation. Ann. Neurol. 72, 610–624, doi: 10.1002/ana.23627 (2012). 23109155

[b29] Noda H., Takeuchi H., Mizuno T. & Suzumura A. Fingolimod phosphate promotes the neuroprotective effects of microglia. J. Neuroimmunol. 256, 13–18, doi: 10.1016/j.jneuroim.2012.12.005 (2013). 23290828

[b30] Cipriani R., Chara J. C., Rodriguez-Antiguedad A. & Matute C. FTY720 attenuates excitotoxicity and neuroinflammation. J Neuro inflammation 12, 86, doi: 10.1186/s12974-015-0308-6 (2015). PMC4429813 25953296

[b31] Doi Y. . Fingolimod phosphate attenuates oligomeric amyloid beta-induced neurotoxicity via increased brain-derived neurotrophic factor expression in neurons. PLoS One 8, e61988, doi: 10.1371/journal.pone.0061988 (2013). 23593505 PMC3625222

[b32] Ruiz A. . Testing Abeta toxicity on primary CNS cultures using drug-screening microfluidic chips. Lab Chip 14, 2860–2866, doi: 10.1039/c4lc00174e (2014). 24914747

[b33] Harada J., Foley M., Moskowitz M. A. & Waeber C. Sphingosine-1-phosphate induces proliferation and morphological changes of neural progenitor cells. J Neurochem 88, 1026–1039 (2004). 14756825 10.1046/j.1471-4159.2003.02219.x

[b34] Kempf A. . The sphingolipid receptor S1PR2 is a receptor for Nogo-a repressing synaptic plasticity. PLoS Biol 12, e1001763, doi: 10.1371/journal.pbio.1001763 (2014). 24453941 PMC3891622

[b35] Choi J. W. & Chun J. Lysophospholipids and their receptors in the central nervous system. Biochim Biophys Acta 1831, 20–32, doi: 10.1016/j.bbalip.2012.07.015 (2013). 22884303 PMC3693945

[b36] Wei Y. . Fingolimod provides long-term protection in rodent models of cerebral ischemia. Ann Neurol 69, 119–129, doi: 10.1002/ana.22186 (2011). 21280082 PMC3200194

[b37] Kraft P. . FTY720 ameliorates acute ischemic stroke in mice by reducing thrombo-inflammation but not by direct neuroprotection. Stroke 44, 3202–3210, doi: 10.1161/STROKEAHA.113.002880 (2013). 24029635

[b38] Hasegawa Y., Suzuki H., Sozen T., Rolland W. & Zhang J. H. Activation of sphingosine 1-phosphate receptor-1 by FTY720 is neuroprotective after ischemic stroke in rats. Stroke 41, 368–374, doi: 10.1161/STROKEAHA.109.568899 (2010). 19940275 PMC2811754

[b39] Lee K. D. . FTY720 reduces inflammation and promotes functional recovery after spinal cord injury. J Neurotrauma 26, 2335–2344, doi: 10.1089/neu.2008.0840 (2009). 19624262 PMC2850297

[b40] Deogracias R. . Fingolimod, a sphingosine-1 phosphate receptor modulator, increases BDNF levels and improves symptoms of a mouse model of Rett syndrome. Proc. Natl. Acad. Sci. USA 109, 14230–14235, doi: 10.1073/pnas.1206093109 (2012). 22891354 PMC3435172

[b41] Gao F. . Fingolimod (FTY720) inhibits neuroinflammation and attenuates spontaneous convulsions in lithium-pilocarpine induced status epilepticus in rat model. Pharmacol Biochem Behav 103, 187–196, doi: 10.1016/j.pbb.2012.08.025 (2012). 22960129

[b42] Takasugi N. . FTY720/fingolimod, a sphingosine analogue, reduces amyloid-beta production in neurons. PLoS One 8, e64050, doi: 10.1371/journal.pone.0064050 (2013). 23667698 PMC3646787

[b43] Fukumoto K. . Fingolimod increases brain-derived neurotrophic factor levels and ameliorates amyloid beta-induced memory impairment. Behav Brain Res 268, 88–93, doi: 10.1016/j.bbr.2014.03.046 (2014). 24713151

[b44] Hemmati F. . Neurorestorative effect of FTY720 in a rat model of Alzheimer’s disease: comparison with memantine. Behav Brain Res 252, 415–421, doi: 10.1016/j.bbr.2013.06.016 (2013). 23777795

[b45] Asle-Rousta M., Kolahdooz Z., Oryan S., Ahmadiani A. & Dargahi L. FTY720 (fingolimod) attenuates beta-amyloid peptide (Abeta42)-induced impairment of spatial learning and memory in rats. J Mol Neurosci 50, 524–532, doi: 10.1007/s12031-013-9979-6 (2013). 23435938

[b46] Lahiri S. . Ceramide synthesis is modulated by the sphingosine analog FTY720 via a mixture of uncompetitive and noncompetitive inhibition in an Acyl-CoA chain length-dependent manner. J Biol Chem 284, 16090–16098, doi: 10.1074/jbc.M807438200 (2009). 19357080 PMC2713526

[b47] Autry A. E. & Monteggia L. M. Brain-derived neurotrophic factor and neuropsychiatric disorders. Pharmacol Rev 64, 238–258, doi: 10.1124/pr.111.005108 (2012). 22407616 PMC3310485

[b48] Jankowsky J. L. . Mutant presenilins specifically elevate the levels of the 42 residue beta-amyloid peptide *in vivo*: evidence for augmentation of a 42-specific gamma secretase. Hum Mol Genet 13, 159–170, doi: 10.1093/hmg/ddh019 (2004). 14645205

[b49] Zhang W. . Soluble Abeta levels correlate with cognitive deficits in the 12-month-old APPswe/PS1dE9 mouse model of Alzheimer’s disease. Behav Brain Res 222, 342–350, doi: 10.1016/j.bbr.2011.03.072 (2011). 21513747

[b50] Kim H. Y. . Taurine in drinking water recovers learning and memory in the adult APP/PS1 mouse model of Alzheimer’s disease. Scientific reports 4, 7467, doi: 10.1038/srep07467 (2014). 25502280 PMC4264000

[b51] Webster S. J., Bachstetter A. D. & Van Eldik L. J. Comprehensive behavioral characterization of an APP/PS-1 double knock-in mouse model of Alzheimer’s disease. Alzheimer’s research & therapy 5, 28, doi: 10.1186/alzrt182 (2013). PMC3706792 23705774

[b52] Di Menna L. . Fingolimod protects cultured cortical neurons against excitotoxic death. Pharmacol Res 67, 1–9, doi: 10.1016/j.phrs.2012.10.004 (2013). 23073075

[b53] Koide Y., Hasegawa T. & Takahashi A. Development of novel EDG3 antagonists using a 3D database search and their structure-activity relationships. Journal of medicinal …, doi: 10.1021/jm020080c (2002). 12361389

[b54] Pyne N. J. & Pyne S. Selectivity and specificity of sphingosine 1-phosphate receptor ligands:“off-targets” or complex pharmacology? Frontiers in pharmacology (2011). 10.3389/fphar.2011.00026 PMC3108476 21687518

[b55] Brunkhorst R., Vutukuri R. & Pfeilschifter W. Fingolimod for the treatment of neurological diseases-state of play and future perspectives. Front Cell Neurosci 8, 283, doi: 10.3389/fncel.2014.00283(2014). 25309325 PMC4162362

[b56] Martins I. C. . Lipids revert inert Abeta amyloid fibrils to neurotoxic protofibrils that affect learning in mice. Embo J 27, 224–233, doi: 10.1038/sj.emboj.7601953 (2008). 18059472 PMC2206134

[b57] Hong S. . Soluble Abeta oligomers are rapidly sequestered from brain ISF *in vivo* and bind GM1 ganglioside on cellular membranes. Neuron 82, 308–319, doi: 10.1016/j.neuron.2014.02.027 (2014). 24685176 PMC4129520

[b58] Stravalaci M., Beeg M., Salmona M. & Gobbi M. Use of surface plasmon resonance to study the elongation kinetics and the binding properties of the highly amyloidogenic Abeta (1–42) peptide, synthesized by depsi-peptide technique. Biosens Bioelectron 26, 2772–2775, doi: 10.1016/j.bios.2010.10.038 (2011). 21112205

[b59] Stravalaci M. . Specific recognition of biologically active amyloid-beta oligomers by a new surface plasmon resonance-based immunoassay and an *in vivo* assay in Caenorhabditis elegans. J Biol Chem 287, 27796–27805, doi: 10.1074/jbc.M111.334979 (2012). 22736768 PMC3431664

[b60] Alberdi E. . Amyloid beta oligomers induce Ca^2+^ dysregulation and neuronal death through activation of ionotropic glutamate receptors. Cell Calcium 47, 264–272, doi: 10.1016/j.ceca.2009.12.010 (2010). 20061018

[b61] Bengtson C. P., Dick O. & Bading H. A quantitative method to assess extrasynaptic NMDA receptor function in the protective effect of synaptic activity against neurotoxicity. BMC Neurosci 9, 11, doi: 10.1186/1471-2202-9-11 (2008). 18218077 PMC2267199

[b62] Gardoni F. . Hippocampal synaptic plasticity involves competition between Ca^2+^/calmodulin-dependent protein kinase II and postsynaptic density 95 for binding to the NR2A subunit of the NMDA receptor. J Neurosci 21, 1501–1509 (2001). 11222640 10.1523/JNEUROSCI.21-05-01501.2001 PMC6762931

[b63] Kopec C. D., Li B., Wei W., Boehm J. & Malinow R. Glutamate receptor exocytosis and spine enlargement during chemically induced long-term potentiation. J Neurosci 26, 2000–2009, doi: 10.1523/JNEUROSCI.3918-05.2006 (2006). 16481433 PMC6674938

[b64] von Engelhardt J., Doganci B., Seeburg P. H. & Monyer H. Synaptic NR2A- but not NR2B-Containing NMDA Receptors Increase with Blockade of Ionotropic Glutamate Receptors. Front Mol Neurosci 2, 19, doi: 10.3389/neuro.02.019.2009 (2009). 19893758 PMC2773170

[b65] Cerpa W., Ramos-Fernandez E. & Inestrosa N. C. Modulation of the NMDA Receptor Through Secreted Soluble Factors. Mol Neurobiol, doi: 10.1007/s12035-014-9009-x (2014). 25429903

[b66] Prybylowski K. . The synaptic localization of NR2B-containing NMDA receptors is controlled by interactions with PDZ proteins and AP-2. Neuron 47, 845–857, doi: 10.1016/j.neuron.2005.08.016 (2005). 16157279 PMC1350965

[b67] Lee H. K. Synaptic plasticity and phosphorylation. Pharmacol Ther 112, 810–832, doi: 10.1016/j.pharmthera.2006.06.003 (2006). 16904750 PMC2748765

[b68] Chen B. S. & Roche K. W. Regulation of NMDA receptors by phosphorylation. Neuropharmacology 53, 362–368, doi: 10.1016/j.neuropharm.2007.05.018 (2007). 17644144 PMC2001266

[b69] Hardingham G. E., Fukunaga Y. & Bading H. Extrasynaptic NMDARs oppose synaptic NMDARs by triggering CREB shut-off and cell death pathways. Nat Neurosci 5, 405–414, doi: 10.1038/nn835 (2002). 11953750

[b70] Autry A. E. . NMDA receptor blockade at rest triggers rapid behavioural antidepressant responses. Nature 475, 91–95, doi: 10.1038/nature10130 (2011). 21677641 PMC3172695

[b71] Nosyreva E. . Acute suppression of spontaneous neurotransmission drives synaptic potentiation. J Neurosci 33, 6990–7002, doi: 10.1523/JNEUROSCI.4998-12.2013 (2013). 23595756 PMC3661220

[b72] Bliss T. V. & Collingridge G. L. Expression of NMDA receptor-dependent LTP in the hippocampus: bridging the divide. Mol Brain 6, 5, doi: 10.1186/1756-6606-6-5 (2013). 23339575 PMC3562207

[b73] Shipton O. A. & Paulsen O. GluN2A and GluN2B subunit-containing NMDA receptors in hippocampal plasticity. Philos Trans R Soc Lond B Biol Sci 369, 20130163, doi: 10.1098/rstb.2013.0163 (2014). 24298164 PMC3843894

[b74] Corradini I. . Epileptiform activity and cognitive deficits in SNAP-25 (+/−) mice are normalized by antiepileptic drugs. Cereb Cortex 24, 364–376, doi: 10.1093/cercor/bhs316 (2014). 23064108

[b75] Braida D. . Cognitive function in young and adult IL (interleukin)-6 deficient mice. Behav Brain Res 153, 423–429, doi: 10.1016/j.bbr.2003.12.018 (2004). 15265638

[b76] Deacon R. M. Assessing nest building in mice. Nat Protoc 1, 1117–1119, doi: 10.1038/nprot.2006.170 (2006). 17406392

[b77] Okabe M., Ikawa M., Kominami K., Nakanishi T. & Nishimune Y. ‘Green mice’ as a source of ubiquitous green cells. FEBS Lett 407, 313–319 (1997). 9175875 10.1016/s0014-5793(97)00313-x

[b78] Klein W. L. Abeta toxicity in Alzheimer’s disease: globular oligomers (ADDLs) as new vaccine and drug targets. Neurochem Int 41, 345–352 (2002). 12176077 10.1016/s0197-0186(02)00050-5

[b79] Beeg M., Stravalaci M., Bastone A., Salmona M. & Gobbi M. A modified protocol to prepare seed-free starting solutions of amyloid-beta (Abeta)(1)(−)(4)(0) and Abeta (1)(−)(4)(2) from the corresponding depsipeptides. Anal Biochem 411, 297–299, doi: 10.1016/j.ab.2010.12.032 (2011). 21185802

[b80] Taniguchi A. . “Click peptide”: pH-triggered *in situ* production and aggregation of monomer Abeta1–42. Chembiochem 10, 710–715, doi: 10.1002/cbic.200800765 (2009). 19222037

[b81] Frassoni C. . Analysis of SNAP-25 immunoreactivity in hippocampal inhibitory neurons during development in culture and *in situ*. Neuroscience 131, 813–823, doi: 10.1016/j.neuroscience.2004.11.042 (2005). 15749336

[b82] Gardoni F. . CaMKII-dependent phosphorylation regulates SAP97/NR2A interaction. J Biol Chem 278, 44745–44752, doi: 10.1074/jbc.M303576200 (2003). 12933808

