# Supplementary material for: Fingolimod Limits Acute Aβ Neurotoxicity and Promotes Synaptic Versus Extrasynaptic NMDA Receptor Functionality in Hippocampal Neurons
Source: Sci Rep. 2017 Jan 30;7:41734. doi: 10.1038/srep41734 (PMC5278353; doi:10.1038/srep41734)
Supplement: Supplementary Material [file srep41734-s1.pdf]

FINGOLIMOD LIMITS ACUTE Aβ NEUROTOXICITY AND PROMOTES SYNAPTIC VERSUS EXTRASYNAPTIC NMDA RECEPTOR FUNCTIONALITY IN HIPPOCAMPAL NEURONS

Pooja Joshi[1], Martina Gabrielli[2], Luisa Ponzoni[3,4], Silvia Pelucchi[5], Matteo Stravalaci[6], Marten Beeg[6], Sonia Mazzitelli[1], Daniela Braida[3], Mariaelvina Sala[2], Enrica Boda[7], Annalisa Buffo[7], Marco Gobbi[6], Fabrizio Gardoni [5], Michela Matteoli[1,2], Elena Marcello[5], Claudia Verderio[1,2]

Correspondence should be addressed to:

Claudia Verderio, CNR Institute of Neuroscience, Via Vanvitelli 32, 20129 Milano, Italy E-mail: c.verderio@in.cnr.it. Phone number: 00390250317011

SUPPLEMENTARY METHODS

Dot Blot Analysis

Hippocampus and cortices of APPswe/PS1dE9 transgenic mice and littermates were homogenized using pasture pipette in 10 volumes of solubilization buffer containing protease inhibitor cocktail. Lysates were centrifuged at 800 × *g* for 10 minutes at 4°C and the supernatant was used to perform dot blot assay after protein quantification. 10μg of protein was spotted in nitrocellulose membrane which was pre-wetted with TBST. Membrane was blocked with 10% milk in 0.01% tween-20 for 1 hr and later incubated with 6E10 (0.1μg/ml) antibody with 5% milk in 0.01% tween-20. After subsequent washes with TBST, membrane was incubated with anti-mouse IgG horseradish peroxidase conjugated antibody for 1 hour. Membrane was further developed by adding detection solution (ECL Western blotting detection reagent from Amersham, UK). The blots were quantified using ImageJ software (National Institutes of Health, Bethesda, MD). Values are presented as arbitrary densitometry units.

ELISA quantification.

Quantitative determination of soluble Aβ 1–42 was performed using ELISA Human A42 Kit (Invitrogen, Paisley PA4 9RF, UK) according to the manufacturer's procedures. Absorbance was detected by 1420 Multilabel Counter Victor 2 (Perkin-Elmer). Hippocampi from APPswe/PS1 mice were homogenated with a tissue homogenizer in 5 volume Triton lysis buffer (Wang et al., 2007b) and centrifugated for 5 minutes at 3,000xg at 4 degrees.

Morphological analysis of dendritic spines.

17 DIV old cultured hippocampal neurons were transfected with dTom using lipofectamine 2000, to delineate the spine morphology. 24hr after transfection neurons were incubated with FTY720 for 1hr and fixed. Images were acquired with a 63x objective using a spinning disk confocal microscope and focal planes were stacked together in a projection. Dendritic length and spine morphology parameters (spine length, head diameter, and neck width) were measured using the ImageJ software (imagej.nhi.gov/ij/). Spines were then classified in categories (mushroom, thin and stubby) based on the morphological parameters according to NeuronStudio software criteria as described in Leonzino et al, 2016.

SUPPLEMENTARY REFERENCES

Leonzino M, Busnelli M, Antonucci F, Verderio C, Mazzanti M, Chini B(2016) The Timing of the Excitatory-to-Inhibitory GABA Switch Is Regulated by the Oxytocin Receptor via KCC2. *Cell Reports* **15**:96-103

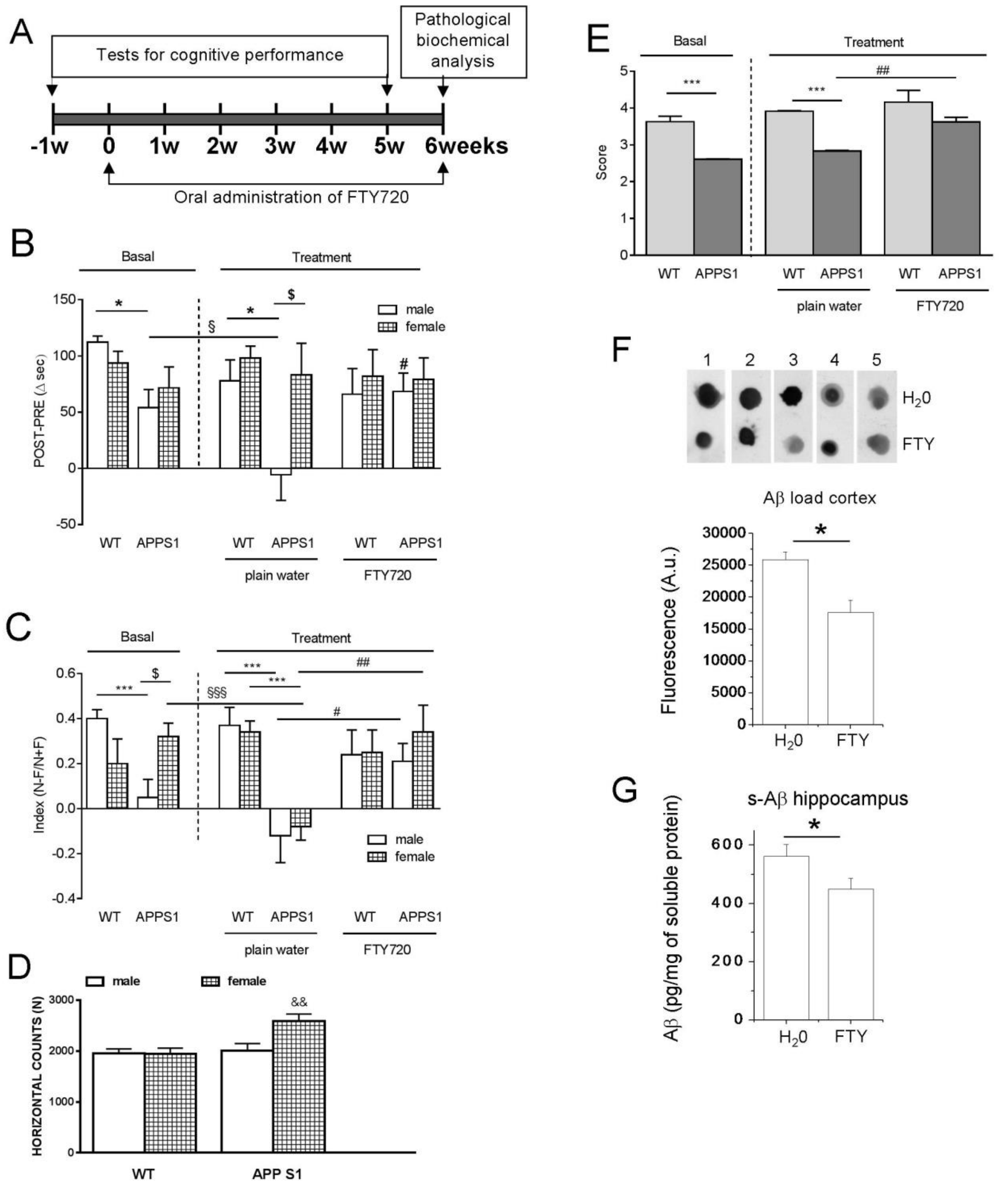

Supplementary Figure 1. **FTY720 improves cognitive impairment in APPswe/PS1dE9 mice**

A Schematic representation of *in vivo* experiments. Cognitive deficits were evaluated 1 week before and 5 weeks after daily administration of FTY720 by oral gavage (1 mg/kg/die) for 5 weeks. At the end of pharmacological treatment mice were sacrificed and subjected to biochemical analyses. **B-E** APPswe/PS1dE9 male mice were impaired in the passive avoidance (**B**) and object recognition (**C**) while female transgenic mice showed cognitive deficit only in the object

recognition test. (**D**) Female mice were slightly hyperactive while male mice had a normal motor activity. (**E**) Both genders were impaired in nesting building. Treatment with drug restored cognitive deficit in the passive avoidance in male APPswe/PS1dE9 mice while it improved cognitive deficit in object recognition and nesting building in both male and female mutant mice (N=10-15 mice/condition; *P<0.05,*** P<0.01 ,vs basal WT, same gender; $ P<0.05, vs corresponding APPS1 male,; § P<0.05,§§§P<0.001 vs corresponding basal APPS1mice;; #P<0.05, ##P<0.01 vs corresponding APPS1, plain water; && P<0.001 vs all the remaining groups (one or Two-way ANOVA, Bonferroni test)). **F** Dot blot assay of total Aβ, probed with 6E10, in cortices from 6 APPswe/PS1dE9 mice treated with plain water or FTY720. Densitometry analysis is shown below (Student's t-test, P=0,006; N=5 mice per condition). **G** Content of s-Aβ in APPswe/PS1dE9 hippocampal samples after treatment with FTY720 or plain water detected by ELISA (N=9 APPswe/PS1dE9 mice/condition; Student's t-test, P=0.011).

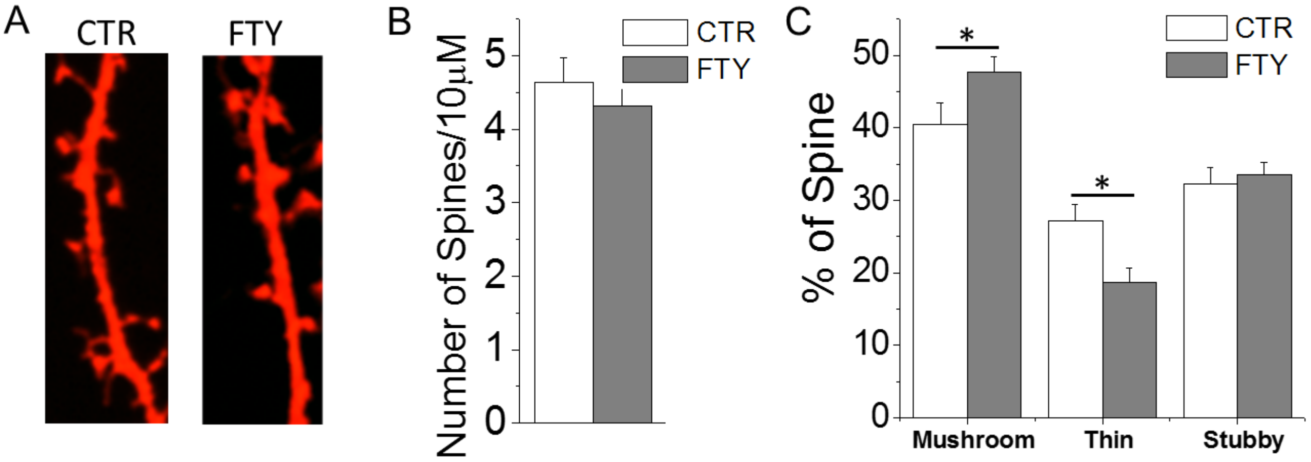

Supplementary Figure 2. FTY20 action on dendritic spines in cultured hippocampal neurons

(**A**) Representative images showing altered spine morphology in dTom (red) positive 18 DIV hippocampal neurons exposed to FTY720 for 1h. (**B**) The histogram shows no changes in the density of dendritic spines in FTY720 –treated neurons. (C) Quantification shows an increase in the percentage of mature spines: mushroom (Student's t-test, P=0.046) and a parallel decrease in immature spines: thin (Student's t-test, P=0.006).
